# Proteolytic signatures define unique thrombin-derived peptides present in human wound fluid *in vivo*

**DOI:** 10.1038/s41598-017-13197-3

**Published:** 2017-10-13

**Authors:** Rathi Saravanan, Sunil S. Adav, Yeu Khai Choong, Mariena J. A. van der Plas, Jitka Petrlova, Sven Kjellström, Siu Kwan Sze, Artur Schmidtchen

**Affiliations:** 1 Lee Kong Chian School of Medicine, Nanyang Technological University, 59 Nanyang Drive, Singapore, 636921 Singapore; 2 Division of Dermatology, Department of Clinical Sciences, Lund University, Lund, Sweden; 3 Centre of Excellence in Biological and Medical Mass Spectrometry “CEBMMS”, Biomedical Centre D13, Lund University, Lund, Sweden; 4 School of Biological Sciences, Nanyang Technological University, 60 Nanyang Drive, Singapore, 637551 Singapore; 5 Wound Healing Centre, Bispebjerg University Hospital, Copenhagen, Denmark

## Abstract

The disease burden of failing skin repair and non-healing ulcers is extensive. There is an unmet need for new diagnostic approaches to better predict healing activity and wound infection. Uncontrolled and excessive protease activity, of endogenous or bacterial origin, has been described as a major contributor to wound healing impairments. Proteolytic peptide patterns could therefore correlate and “report” healing activity and infection. This work describes a proof of principle delineating a strategy by which peptides from a selected protein, human thrombin, are detected and attributed to proteolytic actions. With a particular focus on thrombin-derived C-terminal peptides (TCP), we show that distinct peptide patterns are generated *in vitro* by the human S1 peptidases human neutrophil elastase and cathepsin G, and the bacterial M4 peptidases *Pseudomonas aeruginosa* elastase and *Staphylococcus aureus* aureolysin, respectively. Corresponding peptide sequences were identified in wound fluids from acute and non-healing ulcers, and notably, one peptide, FYT21 (FYTHVFRLKKWIQKVIDQFGE), was only present in wound fluid from non-healing ulcers colonized by *P*. *aeruginosa* and *S*. *aureus*. Our result is a proof of principle pointing at the possibility of defining peptide biomarkers reporting distinct proteolytic activities, of potential implication for improved diagnosis of wound healing and infection.

## Introduction

Conditions, such as arterial and venous insufficiency, diabetes, or excessive pressure all predispose to the formation of non-healing ulcers. In the United States alone, about 6.5 million have chronic skin ulcers^1^. The economic burden on the healthcare system and on society due to failing skin repair and non-healing ulcers is extensive^2^. In diabetic patients, these ulcers are the leading cause (85%) of all major lower limb amputations, inflicting significant pain and reducing quality of life for patients^3^. With a growing diabetic population, chronic wounds affect millions of people, with high morbidity and mortality rates, emerging as a serious threat to healthcare systems around the world^4^. Furthermore, complications of slow or non-healing wounds also affect burn victims. In the United States alone, more than 1.25 million people per year suffer from burns, where local infections cause delayed healing and risk for invasive infections and sepsis. Also notable is that a significant part of hospital antibiotics consumption is related to postoperative prophylaxis, and the costs for postoperative infections are high.

Standard wound diagnostic procedures usually involve clinical evaluations and sometimes bacteriological identification^5^. Occurrence of multidrug resistant microorganisms has further complicated the wound diagnostic procedures and treatment options. In addition, the management of drug resistant microorganisms is in an urgent need for better treatment options^6,7^. Early diagnosis paired with prompt and effective therapeutic interventions in case of infection risk may lead to better treatments targeting the causes of wounds, as well as reduction of wound infections. Clearly, there is an unmet need for improved diagnostic approaches in order to better predict healing activity and infection in non-healing wounds.

Non-healing ulcers are characterized by the presence of an elevated level of proteases, both endogenous and of bacterial origin, which disrupts the delicate balance between acute inflammation and progression of wound healing^8^. Consequently, these wounds are often characterized by high inflammatory activity, excessive proteolytic processing of matrix molecules and immune mediators and impaired wound closure^9–11^. Hemostasis is the initial phase of wound healing and commences with the formation of active thrombin which in turn, mediates fibrin clot formation as well as pro-inflammatory and chemotactic effects^12,13^. We have previously demonstrated that proteolysis of thrombin generates host defense peptides (HDPs), particularly derived from the C-terminal part of the molecule^14^. These thrombin-derived C-terminal peptides (TCPs) bind to bacterial lipopolysaccharide (LPS) and display both anti-microbial and anti-inflammatory activities *in vitro*, with therapeutic potential against infection and septic shock *in vivo* ^15,16^. Interestingly, we found that *Pseudomonas aeruginosa* may mimic the formation of such HDPs, leading to generation of anti-inflammatory peptides and circumvention of host responses^17^. In a recent report, we also demonstrated a previously undisclosed role of larger TCPs of about 11 kDa, involving LPS- and bacteria-induced aggregation and scavenging, facilitating clearance and microbial killing^18^.

Previous studies have mostly dealt with identification of major HDPs of thrombin from a structural, functional, and therapeutic perspective. However, wound fluid proteolytic products, including TCPs, may also serve as a “window” into the wound microenvironment. We therefore hypothesized that the peptide profile could reflect the action of different proteases present in wounds. However, the complexity of such peptide systems, and lack of efficient analytic tools, until recently has hampered their clinical exploitation and use. Here, we set out to define cleaved thrombin fragments generated by both endogenous and bacterial proteases. Our results show that cleavage of thrombin by human and bacterial proteases yields unique peptide patterns. Multiple fragments of TCPs, generated by both human neutrophil and bacterial proteases were identified, and similar TCPs were found in human wound fluid obtained from sterile as well as infected wounds. Taken together, our study demonstrates a proof of principle of the feasibility for defining peptide biomarkers that “report” on distinct protease activities *in vivo*.

## Results

### Degradation of thrombin by endogenous human proteinases *in vitro*

**Figure 1:**
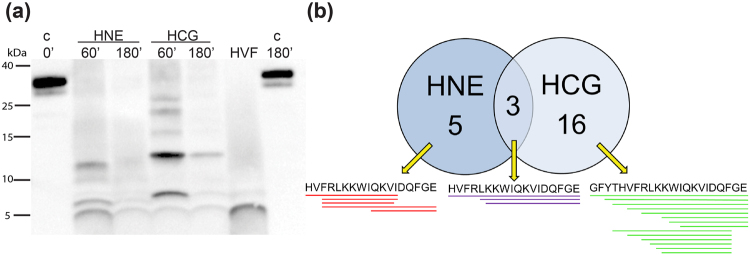
Proteolytic digestion of thrombin by human proteases. (**a**) Western blot analysis of human α-thrombin digested with human neutrophil elastase (HNE) and human cathepsin G (HCG). The samples were incubated for the indicated time periods at an enzyme: substrate ratio of 1:30 (w/w) and antibody against the C-terminal part of thrombin was used for visualization. Thrombin (incubated for 0 and 180 minutes) and the synthetic thrombin C-terminal peptide HVF18 were loaded as control. (**b**) A Venn-diagram representing the LC-MS/MS identified unique and overlapping (purple) TCP peptide sequences generated by HNE (red) and HCG (green) in an experimental repeat of three.

Thrombin susceptibility to endogenous human proteases secreted by neutrophils during wounding was assessed by analyzing the *in vitro* peptide patterns generated by human neutrophil elastase (HNE) and cathepsin G (HCG). Both HNE and HCG cleaved thrombin completely, generating a large number of fragments (157 and 129 peptides respectively) as evident from the SDS-PAGE and mass spectrometric analysis (Figure S1). Peptide sequence comparisons of the LC-MS/MS data identified distinct TCPs, indicating that HNE and HCG cleave thrombin specifically, with low overlap (Fig. 1 and Figure S4). Furthermore, co-incubation of thrombin with HNE and HCG simultaneously did not yield additional common peptide fragments (Figure S2). Interestingly, the peptide HVF18 (HVFRLKKWIQKVIDQFGE), a fragment having anti-endotoxic effects was identified in the peptide products of HNE and HCG, both S1 peptidases (Table 1). The results support earlier reports that HNE cleaves thrombin to release bioactive thrombin-derived peptides^19,20^ including HVF18^14^.

**Table 1:** Sequences of thrombin C-terminal peptides (TCPs) generated by human and bacterial proteases.

Sequences	HNE	HCG	PAE	ALYS	V8
HVFRLKKWIQKVI	**+**				
FRLKKWIQKVIDQFGE	**+**				
FRLKKWIQKVI	**+**				
FRLKKWIQKV	**+**				
QKVIDQFGE	**+**				
GFYTHVFRLKKWIQKVIDQFGE		**+**			
THVFRLKKWIQKVIDQFGE		**+**			
THVFRLKKWIQKVIDQF		**+**			
VFRLKKWIQKVIDQF		**+**			
RLKKWIQKVIDQFGE		**+**			
RLKKWIQKVIDQF		**+**			
LKKWIQKVIDQF		**+**			
KKWIQKVIDQF		**+**			
KWIQKVIDQF		**+**			
WIQKVIDQF		**+**			
IQKVIDQF		**+**			
HVFRLKKWIQKVIDQFGE	**+**	**+**			
KKWIQKVIDQFGE	**+**	**+**			
LKKWIQKVIDQFGE	**+**	**+**	**+**	**+**	**+**
KWIQKVIDQFGE		**+**	**+**	**+**	**+**
FYTHVFRLKKWIQKVIDQFGE			**+**	**+**	
YTHVFRLKKWIQKVIDQFGE		**+**	**+**	**+**	
VFRLKKWIQKVIDQFGE		**+**			**+**
WIQKVIDQFGE		**+**	**+**	**+**	
IQKVIDQFGE		**+**	**+**	**+**	
FYTHVFRLKKWIQKVIDQ			**+**		
FYTHVFRLKKWIQK			**+**		
FYTHVFRLKKW			**+**		
YGFYTHVFR				**+**	
LKKWIQKVIDQ				**+**	

^*^HNE, Human neutrophil elastase; HCG, CathepsinG, PAE, *Pseudomonas aeruginosa* elastase; ALYS, Staphylococcus aureus aureolysin, V8 protease.

### Degradation of thrombin by bacterial proteinases *in vitro*

**Figure 2:**
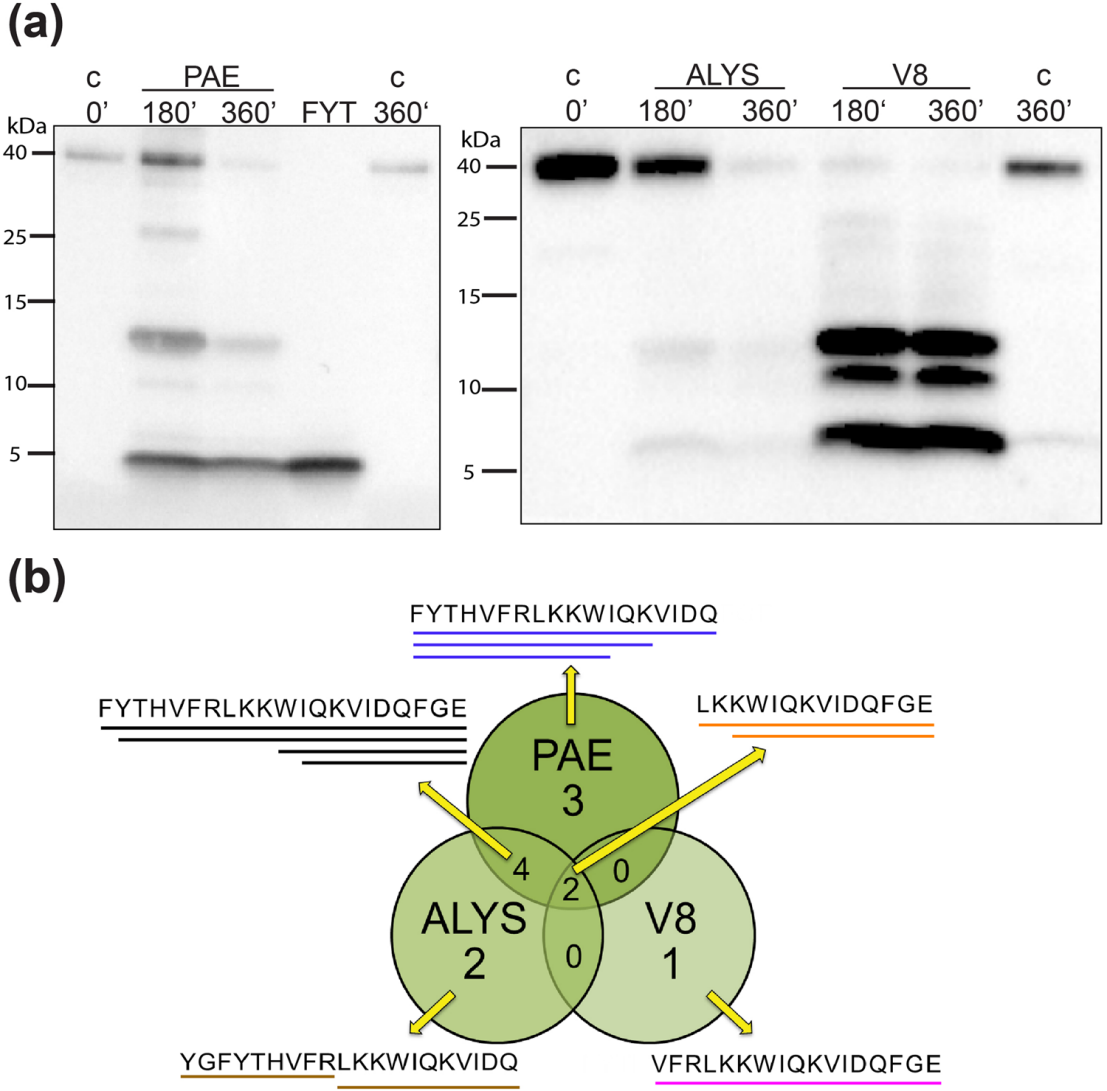
Bacterial proteases cleave human thrombin. (**a**) Western blot analysis of human α-thrombin digested with *P*. *aeruginosa* elastase (PAE), *S*. *aureus* aureolysin (ALYS) and V8 protease, incubated at 37 °C for 3 and 6 hours. Thrombin incubated for 6 hours and the synthetic peptide FYT21 were loaded as control. PAE western blot is shown at exposure time of 20 secs while the V8/ALYS blot is at 8secs. The V8/ALYS western blot at exposure time of 20 secs is provided in the supplementary information S3. (**b**) A Venn-diagram comparing the LC-MS/MS identified TCPs produced by bacterial proteases PAE (blue), ALYS (brown) and V8 (magenta) in an experimental repeat of three, highlights unique and overlapping peptide sequences (black and orange).

**Figure 3:**
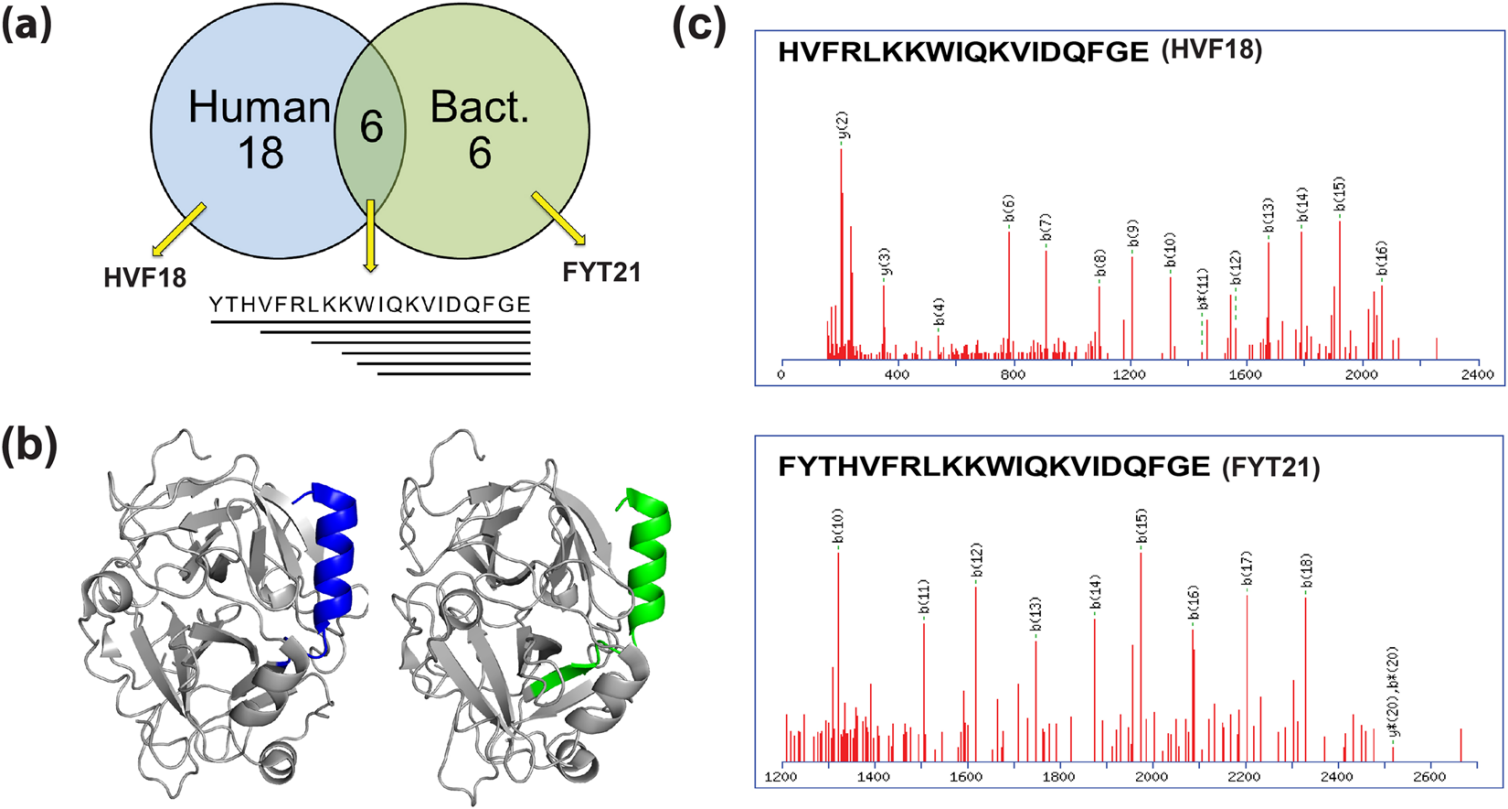
Human and bacterial proteases cleave thrombin to generate unique peptide patterns. (**a**) Comparison of TCPs obtained combining peptides generated by human proteases (HNE and HCG) and bacterial proteases (PAE, ALYS, and V8) highlighting HVF18 and FYT21 as unique products of digestion by human and bacterial protease, respectively. (**b**) 3D structure of thrombin highlighting the neutrophil elastase-cleaved thrombin C-terminal peptide fragment HVF18 (blue) and the *P*. *aeruginosa* elastase-generated peptide FYT21 (green). (**c**) MS/MS spectrum of the respective protease generated peptides HVF18 and FYT21.

**Figure 4:**
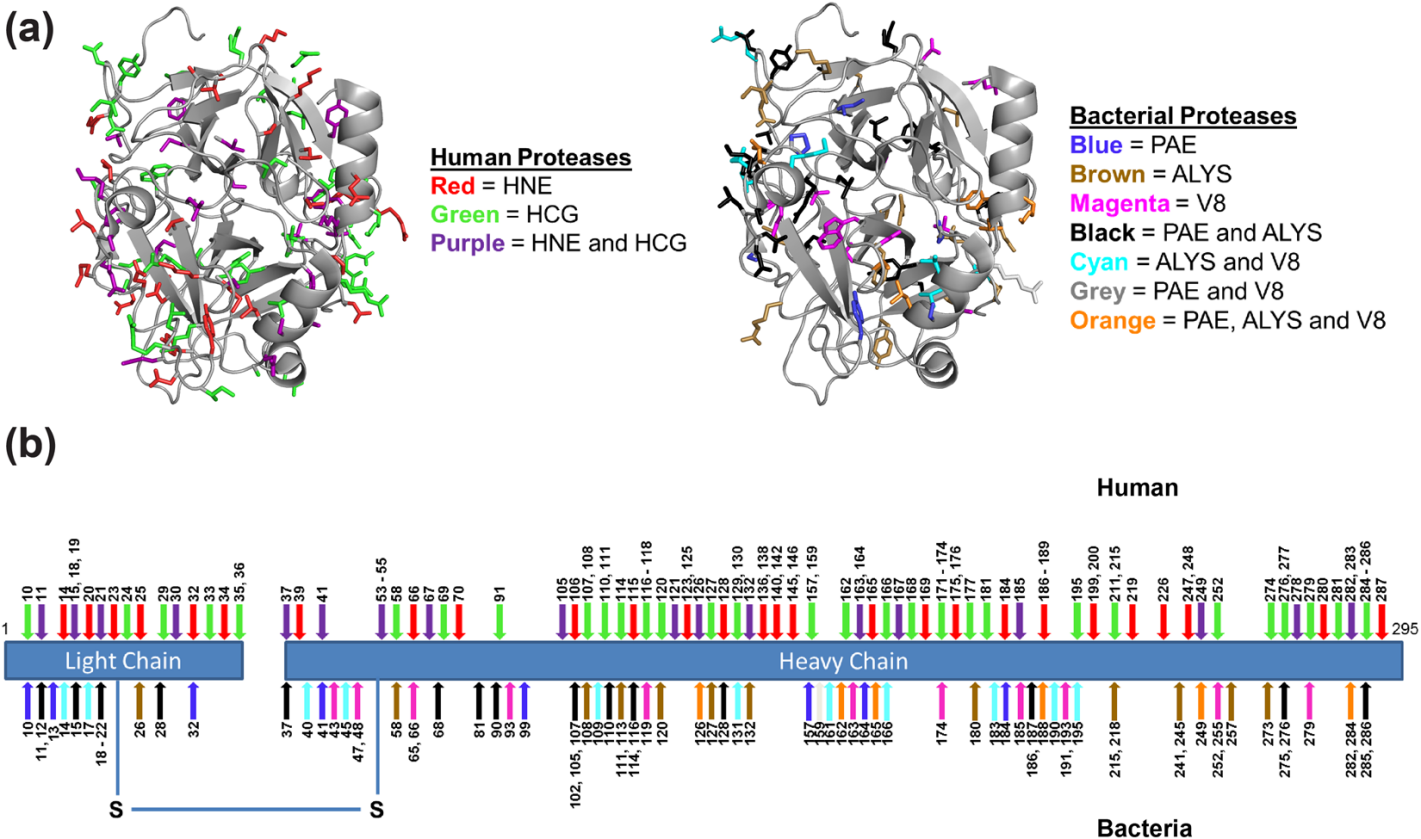
Illustration of protease cleavage sites in human thrombin. (**a**) 3D model showing cleavage sites of human and bacterial proteases highlighting the exterior disposition of enzyme recognition sites in thrombin. (**b**) Color-coded 2D representation of the protease cleavage sites indicating similarities and differences between the human and bacterial proteases.

Next, we analysed the thrombin peptides generated by bacterial proteinases such as elastase of the Gram-negative *Pseudomonas aeruginosa*, and aureolysin and V8 proteases of the Gram-positive *Staphylococcus aureus*, two bacteria commonly found in non-healing ulcers and infected burn wounds (Figure S3). As seen in Fig. 2, *P*. *aeruginosa* elastase (PAE) generates a 21 amino acid TCP, FYT21 (FYTHVFRLKKWIQKVIDQFGE), reported recently to inhibit host inflammatory responses^17^. Notably, FYT21 was also found to be generated by *S*. *aureus* aureolysin (ALYS), a metalloproteinase like *P*. *aeruginosa* elastase (Fig. 2 and Table 1). Peptides generated by V8 were mainly of high molecular weight, in comparison to those generated by PAE and ALYS, possibly reflecting the limited specificity of the protease for anionic residues (D/E) at the P1 position (Figure S4). Hence, a limited number of TCPs were identified after V8 digestion (Figure S3). Taken together, similar to the findings with HNE and HCG, the results indicate that bacterial proteases cleave thrombin distinctly, yielding unique peptide patterns (Fig. 3). Notably, the TCPs HVF18 and FYT21 were identified as unique products of human and bacterial protease activity, respectively (Fig. 3 and Table 1). Figure 4 illustrates the obtained cleavage sites by the respective proteases as shown in a 3D model of thrombin (panel A), as well as indicated in the amino acid sequence (panel B). The side chain dispositions of cleavage sites, as highlighted in the 3D model, indicates that the majority of cleavage sites for both bacterial and human proteases are situated on the exterior surface of thrombin.

### Thrombin-derived peptides are identified in human wound fluids

**Figure 5:**
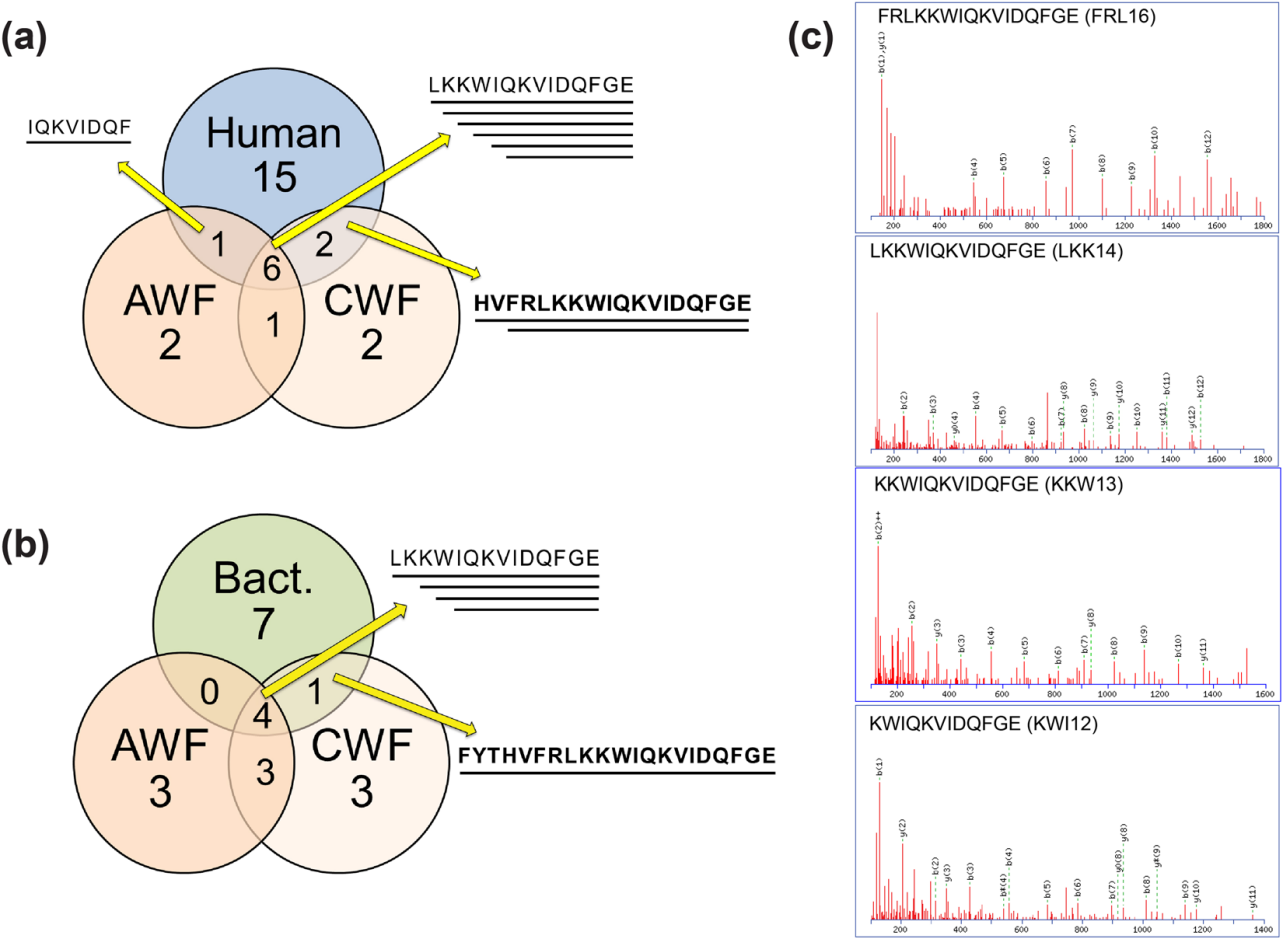
Comparison of *in vitro* and *in vivo* generated thrombin C-terminal fragments. A Venn-diagram showing TCPs generated by (**a**) human proteases and (**b**) bacterial proteases found in human wound fluids from acute wounds (AWF) and non-healing ulcers (CWF). Overlapping peptides identified in wound fluid are indicated by a yellow arrow. HVF18 and FYT21 sequence is bolded. (**c**) MS/MS spectrum of protease generated truncated TCPs FRL16, LKK14, KKW13, and KWI12 found in the wound fluid.

In order to identify TCPs present in human wounds, wound fluids from acute surgical wounds and chronic ulcers co-infected with *P*. *aeruginosa* and *S*. *aureus* were incubated with thrombin C-terminal antibody-coated dynabeads to pull-down TCPs, followed by mass spectrometry analysis. Comparison of the *in vitro* generated peptides and the fragments detected *in vivo* showed the presence of multiple fragments of TCPs in both acute wound fluids (AWF) and chronic wound fluids (CWF) (Fig. 5 and Table 2). The peptides HVF18 and FYT21, products of human and bacterial proteases respectively, were identified in wound fluid from non-healing ulcers, while the peptides FRL16 (FRLKKWIQKVIDQFGE), LKK14 (LKKWIQKVIDQFGE), KKW13 (KKWIQKVIDQFGE) and KWI12 (KWIQKVIDQFGE), and other truncated fragments were common in wound fluids from both acute wounds and non-healing ulcers (Fig. 5 and Table 2).

**Table 2:** Sequences of thrombin C-terminal peptides (TCPs) generated by human and bacterial proteases found in human acute wound (AWF) and chronic wound fluids (CWF).

**Sequences**	**HP**	**BP**	**AWF**	**CWF**
GFYTHVFRLKKWIQKVIDQFGE	**+**			
THVFRLKKWIQKVIDQFGE	**+**			
RLKKWIQKVIDQFGE	**+**			
THVFRLKKWIQKVIDQF	**+**			
VFRLKKWIQKVIDQF	**+**			
RLKKWIQKVIDQF	**+**			
LKKWIQKVIDQF	**+**			
KKWIQKVIDQF	**+**			
KWIQKVIDQF	**+**			
WIQKVIDQF	**+**			
HVFRLKKWIQKVI	**+**			
FRLKKWIQKVI	**+**			
FRLKKWIQKV	**+**			
FYTHVFRLKKWIQKVIDQ		**+**		
FYTHVFRLKKWIQK		**+**		
FYTHVFRLKKW		**+**		
LKKWIQKVIDQ		**+**		
YGFYTHVFR		**+**		
LKKWIQKVIDQFGE	**+**	**+**	**+**	**+**
KWIQKVIDQFGE	**+**	**+**	**+**	**+**
WIQKVIDQFGE	**+**	**+**	**+**	**+**
IQKVIDQFGE	**+**	**+**	**+**	**+**
FYTHVFRLKKWIQKVIDQFGE		**+**		**+**
YTHVFRLKKWIQKVIDQFGE	**+**	**+**		
HVFRLKKWIQKVIDQFGE	**+**			**+**
VFRLKKWIQKVIDQFGE	**+**	**+**		
FRLKKWIQKVIDQFGE	**+**			**+**
KKWIQKVIDQFGE	**+**		**+**	**+**
QKVIDQFGE	**+**		**+**	**+**
KVIDQFGE			**+**	**+**
VIDQFGE				**+**
IDQFGE			**+**	
IQKVIDQFG			**+**	
IQKVIDQF	**+**		**+**	

^*^HP, Human protease; BP, bacterial protease, AWF, acute wound fluid; CWF, chronic wound fluid.

## Discussion

The main finding of this work is the definition of multiple fragments of TCPs which are generated by both human and bacterial proteases. Furthermore, the work shows a proof of principle that it may be technically possible to define peptides “reporting” distinct proteolytic activity patterns not only *in vitro*, but also *in vivo*. As protease activity is so fundamentally linked to the healing status of wounds^9^ as well as the presence of protease-producing bacteria, the work thus points at the future possibility of using peptides as biomarkers in the development of advanced wound and infection diagnostics - based on mass spectrometry technologies, antibody arrays, or other sensor-based technologies.

As mentioned in the introduction, today’s measures in the clinic to assess a wound and define whether it is infected or not, mainly depend on the clinician’s evaluation. In general practice, if there is a suspected infection, a microbiological swab is taken before antibiotics are given to the patient. It is notable that most wound swabs are prone to false positives as most will yield bacterial growth which is not always due to infection. For example, over 90% of non-healing ulcers are colonized by staphylococci - even without infection. The clinical evaluation of infection today is based on obvious signs of infection including redness, heat, swelling, purulent exudate, smell, pain, systemic illness, and the presence of fistulas, “foamy” granulation tissue, or tissue breakdown. The challenge is to detect infection before it reaches this stage.

Alternate methods to swab technique for detecting bacterial infection are based on looking at products from bacteria, or the host, or determining host responses that can report the presence of infection. At current, some developments are directed to the identification of a variety of biochemical by-products. For example, pyocyanin—the blue–green pigment secreted by many *P*. *aeruginosa* strains, has been used to detect bacteria^21^. Urate or uric acid is another metabolite found within wounds, which may be used as a diagnostic marker. The problem here is that bacteria may metabolize urate. Toxins produced by bacteria found in wounds may be used to trigger a sensor output indicating early infection. For example, Zhou *et al*. developed Trojan-like phospholipid vesicles, which release a fluorescent dye cargo when subjected to bacterial toxins^22,23^. Of relevance to this study is that HNE and HCG have recently been reported as early stage warning markers for non-healing ulcers^24^. The enzymatic nature of HNE and HCG has indeed been used to produce a sensor made from a chromophore linked by a short peptide sequence susceptible to cleavage by each of the enzymes. For example, Edwards *et al*. used cellulose-AP-suc-Ala-Ala-Pro-Ala-pNA substrates^25^. Rimmer *et al*. developed a method for binding either Gram-positive or Gram-negative bacteria to a polymer which alters shape upon binding and has potential as a bacterial sensor^26,27^. Matrix metalloproteinases (MMPs) are increased in chronic wound fluid (CWF). Gao *et al*. sensed MMP-2 at concentrations as low as 0.1 ng/mL by spin-coating a gelatin film on top of a pSi resonator^28^. Upon contact with MMP-2, the film was degraded and the generated peptides were able to enter the pores and induced color changes that could be detected by the eye.

Other researchers are focusing on protein patterns, and identification of the total proteome of wounds has been suggested as one method to obtain not only information of the pathogenesis, but also to be able to define specific protein biomarkers discriminating between various wound types^29–31^. For example, Eming *et al*. demonstrated the differential distribution of tissue repair proteins in exudates of healing wounds and persistent inflammatory tissue damaging mediators in non-healing wounds^24^. Upton’s group has established enhanced proteomics workflow to reduce sample complexity by selective depletion of high abundant protein, validating detection of proteins present in low concentrations in chronic wound fluids^32^. Auf dem Keller *et al*. applied quantitative proteomics strategies to dissect proteolytic pathways in healthy and diseased skin. In a global approach, they analysed wound fluids from a porcine vacuum assisted closure (VAC) wound models and quantitatively assessed the wound proteome and the activity of distinct protease groups along the healing process^33^. They also mapped proteolytic pathways *in vivo* and established protease-substrate relations that will help to better understand protease action in cutaneous wound repair^34^. Considering the above, it is of note that many approaches are based on detection of products such as pyocyanin, toxins, or proteases. Methods for detection of down-stream products reflecting inflammation are rare. Of note is that auf dem Keller´s work involves studies on such protease patterns. However, as their method focuses on “N-terminomics”, low molecular weight peptides are mainly lost during the preparation steps. Notable are also recent developments, where a labelling approach^35^ using trimethoxyphenyl phosphonium (TMPP) allows characterization of both N-terminal and internal digestion peptides.

Whether the here reported method based on peptide detection will reach a sufficient sensitivity and specificity in order to be of diagnostic use has to be evaluated in future studies. For example, although a recently developed assay, Woundchek™ was shown to have a good sensitivity for detection of HNE and MMP levels, the predictive potential of protease measurements for various wound pathologies, although showing promising results^36^, needs further clinical studies. From this perspective, it is therefore of note that some peptide fragments were only produced by bacterial enzymes, such as the TCP FYT21. Hence, using peptide markers that are unique for bacteria should enable a high specificity, since such peptides should not be detected at all in sterile wounds. An important factor to address in future studies, of importance for evaluating the diagnostic utility of FYT21 and related fragments, is their stability and half-life *in vivo*. Of interest is that recent work by Böttger *et al*.^37^ showed peptides with differing cleavage sites exhibited variable proteolytic sensitivities in blood, serum, and plasma. Therefore, future developments aiming at defining peptides as biomarkers must be preceeded by a careful analysis of their potential degradation (e.g. using isotope-labelled peptides), particularly in relevant environments, such as wound fluids from acute or chronic wounds.

It is also of note that *P*. *aeruginosa* elastase belongs to a superfamily of zinc-containing metallopeptidases of the M4 (thermolysin) class, also secreted from bacteria such as *Enterococcus faecalis* (coccolysin) and *S*. *aureus* (aureolysin), bacteria very common in chronic wounds. The present findings confirmed the presence of FYT21 after digestion with *P*. *aeruginosa* elastase, as previously communicated^17^. Interestingly, we also detected the peptide after digestion with aureolysin, another staphylococcal proteinase of the M4 peptidase group. Hence, it is tempting to speculate that formation of FYT21 could be common for many M4 peptidase-producing bacteria. If so, such peptide markers may reflect bacterial actions more than the presence of bacteria *per se*, which from a physiological point of view should correlate better to overall bacterial load and actions, and ultimately also, the clinical status of a particular wound. Hypothetically, combining the use of specific, qualitative, peptide markers, such as the ones reported here, with Woundchek™-based analyses of HNE and MMPs or other related more quantitative approaches, could be an attractive strategy in order to attain a higher diagnostic sensitivity and specificity with respect to wound status. These evaluations in turn, could be complemented with sensitive bacterial detection methods, such as the ones mentioned above, which altogether should provide a “multi-dimensional” analysis of wound status, enabling simultaneous determination of bacteria *per se*, host proteolytic activities, as well as down-stream specific peptides reporting actions of host or bacterial proteases. Clearly, clinical prospective studies on larger patient groups, where bacterial types and loads, and wound proteolysis levels, are determined and correlated with the occurrence of the herein defined peptide patterns, are mandated in order to fully investigate such potential diagnostic possibilities.

From a biological perspective, our findings also illustrate the multi-functionality of thrombin. Being a major enzyme of the coagulation cascade, thrombin acts as a procoagulant converting soluble fibrinogen into insoluble fibrin to initiate wound repair. However, the biological function of thrombin expands beyond blood coagulation, as the enzyme triggers endothelial cell activation, increased adhesion of neutrophils to the endothelium^38^, platelet aggregation and chemotaxis of neutrophils^39^ and the induction of cytokine release from epithelial and endothelial cells^32,40,41^ through proteolytically activated receptors (PAR1, PAR3 or PAR4). Next, intermediate-sized TCPs similar to the B4 chain of γ-thrombin are subsequently formed, enabling aggregation and phagocytosis of bacteria^18^. Finally, small TCPs, such as FYT21 and HVF18, generated by proteolysis can block pro-inflammatory cytokine responses via binding to LPS. Apart from these fragments, our data shows that a multitude of truncated TCP variants seem to be generated, and it is tempting to speculate that such forms could have additional biological functions, or work in synergy with larger TCP fragments. In this context, it is interesting that other bioactive thrombin-related fragments, such as the peptides ELLESYIDGR and TATSEYQTFFNPR, are identified in platelet rich plasma extracts and promote angiogenesis and wound healing *in vitro* ^42^, findings exploited in the design of hybrid peptides for improved wound healing *in vivo* ^42,43^. Another peptide, AGYKPDEGKRGDACEGDSGGPFV, derived from the receptor-binding part of thrombin accelerates wound repair^44^. Notable is that we identified truncated forms related to the ELLESYIDGR sequence in our *in vitro* protease digests, whereas the sequence AGYKPDEGKRGDACEGDSGGPFV was included in longer fragments produced *in vitro* by HNE and ALYS (Table S4). Hence, looking beyond TCPs, it is also possible that future detailed analyses of other thrombin sequences could provide interesting clues to possible relationships between levels of such peptides in wounds and different wound healing states. Taken together, the current report, here mainly focusing on TCPs, is a proof of principle showing that unique peptide patterns can be used for definition of protease actions, of possible use in future diagnostic developments.

## Materials and Methods

### Peptides and proteins

Thrombin was from Innovative Research, USA. The peptides FYT21 (FYTHVFRLKKWIQKVIDQFGE) and HVF18 (HVFRLKKWIQKVIDQFGE), were synthesized by Biopeptide (San Diego, CA). The purity of these peptides (>95%) was confirmed with MALDI-TOF MS. The endoproteinases, human neutrophil elastase and cathepsin G were from Calbiochem, USA. V8 Glu-C endoproteinase was obtained from Roche, aureolysin from BioCol, and *P*. *aeruginosa* elastase (PAE) was purified as previously described with some modifications^45^.

### Biological materials

Wound fluids from non-healing venous ulcers co-infected with *P*. *aeruginosa* and *S*. *aureus* were collected under a Tegaderm dressing for 2 hours as reported earlier^46^. Bacteria were identified by routine bacteriological methods from swabs from the wound surface. The patients were not treated with antibiotics. Sterile wound fluids were obtained from surgical drainages after mastectomy. Collection was for 24 to 48 hours after surgery. Wound fluids were centrifuged, aliquoted and stored at −20 °C. The use of human wound fluids was approved by the Ethics Committee at Lund University (LU 708-01 and LU 509-01). All experimental methods were performed in compliance to the guidelines and regulations of the Ethics Committee at Lund University, Lund Sweden with written informed consent from all participants.

### Digestion by human proteases *in vitro*

Thrombin (4 μg–15 μg) was incubated with either human neutrophil elastase (HNE) or human neutrophil cathepsin G (HCG) at an enzyme-substrate ratio of 1:30 (w/w) in 10 mM Tris, pH 7.4 (volume of 10 μl) at 37 °C for variable time points (30, 60 and 180 minutes). The enzyme activity was blocked after the incubation by heating the samples at 95 °C for 3 minutes and further analysed by Tricine SDS-PAGE. The *in vitro* digestion analysis was performed in triplicates.

### *In vitro* digestion by bacterial proteases

Thrombin (4 μg–15 μg) was incubated at 37 °C with either V8 protease (0.2 μg/μg of thrombin), aureolysin (0.2 μg/μg of thrombin) or PAE (4 mU/μg of thrombin) separately in 10 mM NH_4_HCO_3_ (pH 7.8), 10 mM Tris containing 5 mM CaCl_2_ (pH 7.8) and 10 mM Tris (pH 7.4) respectively in a total volume of 10 μl for 3 hours and 6 hours. The enzyme activity was blocked by heating the sample at 95 °C for 3 minutes and analysed by Tricine SDS-PAGE. Digestion analysis was performed in triplicates.

### SDS-PAGE and silver staining

Seven μl of Novex sample buffer was added to each sample, and heated at 95 °C for 10 minutes. The peptides, FYT21 and HVF18 were loaded for comparison. The samples were analysed by SDS-PAGE using a 10–20% Novex Tricine pre-cast gel run at 90 V, for 2 hours. The gel was stained with a Pierce™ Silver Stain Kit according to the manufacturer’s protocol.

### Immunoblotting

Immediately following SDS-gel electrophoresis, the gels were assembled into the blot module (Invitrogen) based on the manufacturer’s protocol. The transfer was performed at 25 V for 90 minutes on ice. After the transfer, the PVDF membranes were blocked with 5% milk for an hour, followed by overnight incubation of polyclonal antibodies recognizing the peptide VFR17 (VFRLKKWIQKVIDQFGE) to a 1:5000 dilution at 4 °C. The membranes were washed 3 times for 10 minutes and incubated with HRP-conjugated secondary antibodies (Dako) at 1:10000 dilutions for an hour. The membranes were washed 3 times for 10 minutes. The C-terminal thrombin fragments were detected by SuperSignal West Dura Extended Duration Substrate (ThermoFisher Scientific, USA) for 5 minutes and thereafter imaged using a gel documentation system (Gel Doc XR + System).

### LC-MS/MS and data analysis

After *in vitro* enzymatic digestion of thrombin, the reaction was stopped by acidifying the samples. The peptides were then dried and reconstituted in 0.1% formic acid (FA). Peptides were separated and analysed on a Dionex Ultimate 3000 RSLC nanoLC system coupled to a Q-Exactive tandem mass spectrometer (Thermo Fisher, MA). Five μl of sample was injected into an acclaim peptide trap column via the auto-sampler of the Dionex RSLC nanoLC system. Flow rate was at 300 nl/min, and mobile phase A (0.1% FA in 5% acetonitrile) and mobile phase B (0.1% FA in acetonitrile) were used to establish a 60 minute gradient. Peptides were analysed on a Dionex EASY-spray column (PepMap® C18, 3um, 100 A) using an EASY nanospray source. An electrospray potential was set at 1.5 kV. A full MS scan (350–1600 m/z range) was acquired at a resolution of 70000 at m/z 200, with a maximum ion accumulation time of 100 ms. Dynamic exclusion was set to 30 seconds. Resolution for MS/MS spectra was set to 35,000 at m/z 200. The AGC setting was 1E6 for the full MS scan and 2E5 for the MS2 scan. The 10 most intense ions above a 1000 count threshold were chosen for HCD fragmentation, with a maximum ion accumulation time of 120 milliseconds. An isolation width of 2 Da was used for the MS2 scan. Single and unassigned charged ions were excluded from MS/MS. For HCD, normalized collision energy was set to 28. The underfill ratio was defined as 0.1%. Raw data files were converted into the mascot generic file format using Proteome Discoverer version 1.4 (Thermo Electron, Bremen, Germany) with the MS2 spectrum processor for de-isotoping the MS/MS spectra. The concatenated target-decoy UniProt human database (total sequences 92867, total residues 36953354, downloaded on 25 July 2016) was used for data searches. Database search was performed with non-enzyme option using an in-house Mascot server (version 2.4.1, Matrix Science, Boston, MA). Oxidation (M) and deamidation (N and Q) were kept as variable modifications. A total of three samples with two technical replicates (3 × 2) for each *in vitro* digest were analysed by LC-MS/MS. The identified peptides were then sorted on the peptide score and hits with <30 were omitted from analyses. Peptide hits consistently identified in all experimental and technical replicates were considered for qualitative comparison between *in vitro* and *in vivo* samples.

### Pull-down of thrombin C-terminal peptides from wound fluids

Dyanbeads M-280 sheep anti-rabbit IgG (Novex Life Technologies) were used according to the manufacturer’s instructions and as per the methods described earlier^17^. Briefly, 100 μl of dynabeads pre-coated with sheep anti rabbit IgG antibodies were coupled with rabbit polyclonal antibodies (50 μl) recognizing the peptide sequence VFR17 (VFRLKKWIQKVIDQFGE) (Innovagen, Lund, Sweden) by incubation overnight at 4 °C. The coated dynabeads were subsequently washed according to the manufacturer´s protocol and incubated with 100 μl of AWF and/or CWF for 1 hour at RT, which was followed by elution with 20 μl of citric acid monohydrate (100 mM, pH 2).

### Mass spectrometry analysis of wound fluid

Acute (AWF) and chronic wound fluids (CWF) from two patients each (n = 2 for AWF and CWF) were utilised in this study. Wound fluid samples were acidified and analysed by online nanoflow liquid chromatography coupled to tandem mass spectrometry (LC-MS/MS). LC-MS/MS experiments were performed on an EASY-nLC system (Thermo Scientific) connected to a LTQ orbitrap Velos Pro (Thermo Scientific) through a nanoelectrospray ion source and analysed as described earlier^17^.

### Data availability

All data generated or analysed during this study are included in this published article (and its Supplementary Information files). The mass spectrometry proteomics data have been deposited to the ProteomeXchange^47^ Consortium via the PRIDE^48^ partner repository with the dataset identifier PXD006992.

## Electronic supplementary material

Supplementary Information

## References

[1] Brigham PA, McLoughlin E (1996). Burn incidence and medical care use in the United States: estimate, trends, and data sources. The Journal of Burn Care and Rehabilitation.

[2] Singer AJ, Clark RA (1999). Cutaneous wound healing. The New England journal of medicine.

[3] Vinh DC, Embil JM (2005). Rapidly progressive soft tissue infections. The Lancet. Infectious diseases.

[4] Chen WY, Rogers AA (2007). Recent insights into the causes of chronic leg ulceration in venous diseases and implications on other types of chronic wounds. Wound repair and regeneration: official publication of the Wound Healing Society [and] the European Tissue Repair Society.

[5] Giacometti A (2000). Epidemiology and microbiology of surgical wound infections. Journal of clinical microbiology.

[6] Neu HC (1992). The crisis in antibiotic resistance. Science.

[7] Taubes G (2008). The bacteria fight back. Science.

[8] Trengove NJ (1999). Analysis of the acute and chronic wound environments: the role of proteases and their inhibitors. Wound repair and regeneration: official publication of the Wound Healing Society [and] the European Tissue Repair Society.

[9] Agren MS (2000). Causes and effects of the chronic inflammation in venous leg ulcers. Acta dermato-venereologica. Supplementum.

[10] Herrick S (1997). Up-regulation of elastase in acute wounds of healthy aged humans and chronic venous leg ulcers are associated with matrix degradation. Laboratory investigation; a journal of technical methods and pathology.

[11] Yager DR, Nwomeh BC (1999). The proteolytic environment of chronic wounds. Wound repair and regeneration: official publication of the Wound Healing Society [and] the European Tissue Repair Society.

[12] Chen LB, Buchanan JM (1975). Mitogenic activity of blood components. I. Thrombin and prothrombin. Proceedings of the National Academy of Sciences of the United States of America.

[13] Carney DH, Cunningham DD (1978). Role of specific cell surface receptors in thrombin-stimulated cell division. Cell.

[14] Papareddy P (2010). Proteolysis of human thrombin generates novel host defense peptides. PLoS pathogens.

[15] Kalle M (2012). Host defense peptides of thrombin modulate inflammation and coagulation in endotoxin-mediated shock and Pseudomonas aeruginosa sepsis. PloS one.

[16] Hansen FC (2015). The Thrombin-Derived Host Defense Peptide GKY25 Inhibits Endotoxin-Induced Responses through Interactions with Lipopolysaccharide and Macrophages/Monocytes. Journal of immunology.

[17] van der Plas MJ (2016). Pseudomonas aeruginosa elastase cleaves a C-terminal peptide from human thrombin that inhibits host inflammatory responses. Nature communications.

[18] Petrlova, J. *et al*. Aggregation of thrombin-derived C-terminal fragments - a novel host defense mechanism. *PNAS* in press (2017). 10.1073/pnas.1619609114 PMC5448181 28473418

[19] Brower MS, Walz DA, Garry KE, Fenton JW (1987). Human neutrophil elastase alters human alpha-thrombin function: limited proteolysis near the gamma-cleavage site results in decreased fibrinogen clotting and platelet-stimulatory activity. Blood.

[20] Bar-Shavit R (1991). An Arg-Gly-Asp sequence within thrombin promotes endothelial cell adhesion. The Journal of cell biology.

[21] Sharp D, Gladstone P, Smith RB, Forsythe S, Davis J (2010). Approaching intelligent infection diagnostics: Carbon fibre sensor for electrochemical pyocyanin detection. Bioelectrochemistry.

[22] Zhou J, Loftus AL, Mulley G, Jenkins AT (2010). A thin film detection/response system for pathogenic bacteria. Journal of the American Chemical Society.

[23] Zhou J (2011). Development of a prototype wound dressing technology which can detect and report colonization by pathogenic bacteria. Biosensors & bioelectronics.

[24] Eming SA (2010). Differential proteomic analysis distinguishes tissue repair biomarker signatures in wound exudates obtained from normal healing and chronic wounds. Journal of proteome research.

[25] Edwards JV, Caston-Pierre S, Howley PS, Condon BD, Arnold JW (2008). A Bio-Sensor for Human Neutrophil Elastase Employs A Bio-Sensor for Human Neutrophil Elastase Employs Peptide-p-nitroanilide Cellulose Conjugates. Sensor Letters.

[26] Sarker P (2011). Highly branched polymers with polymyxin end groups responsive to Pseudomonas aeruginosa. Biomacromolecules.

[27] Shepherd J (2010). Binding bacteria to highly branched poly(N-isopropyl acrylamide) modified with vancomycin induces the coil-to-globule transition. Journal of the American Chemical Society.

[28] Gao L, Mbonu N, Cao L, Gao D (2008). Label-free colorimetric detection of gelatinases on nanoporous silicon photonic films. Analytical chemistry.

[29] Cravatt BF, Simon GM, Yates JR (2007). The biological impact of mass-spectrometry-based proteomics. Nature.

[30] Broadbent J, Walsh T, Upton Z (2010). Proteomics in chronic wound research: potentials in healing and health. Proteomics. Clinical applications.

[31] Yager DR, Zhang LY, Liang HX, Diegelmann RF, Cohen IK (1996). Wound fluids from human pressure ulcers contain elevated matrix metalloproteinase levels and activity compared to surgical wound fluids. The Journal of investigative dermatology.

[32] Kranzhofer R (1996). Thrombin potently stimulates cytokine production in human vascular smooth muscle cells but not in mononuclear phagocytes. Circulation research.

[33] Sabino F (2015). *In vivo* assessment of protease dynamics in cutaneous wound healing by degradomics analysis of porcine wound exudates. Molecular & cellular proteomics: MCP.

[34] Pascal Schlage, T. K., Jayachandran N. Kizhakkedathu and Ulrich auf dem Keller. Monitoring matrix metalloproteinase activity at the epidermal–dermal interface by SILAC-iTRAQ-TAILS. *Proteomics* **15**, 2491–2502 (2015). 10.1002/pmic.201400627 25871442

[35] Westermann B, Jacome ASV, Rompais M, Carapito C, Schaeffer-Reiss C (2017). Doublet N-Terminal Oriented Proteomics for N-Terminomics and Proteolytic Processing Identification. Methods in molecular biology.

[36] Lazaro JL (2016). Elevated levels of matrix metalloproteinases and chronic wound healing: an updated review of clinical evidence. Journal of wound care.

[37] Bottger R, Hoffmann R, Knappe D (2017). Differential stability of therapeutic peptides with different proteolytic cleavage sites in blood, plasma and serum. PloS one.

[38] Toothill VJ, Van Mourik JA, Niewenhuis HK, Metzelaar MJ, Pearson JD (1990). Characterization of the enhanced adhesion of neutrophil leukocytes to thrombin-stimulated endothelial cells. Journal of immunology.

[39] Bizios R, Lai L, Fenton JW, Malik AB (1986). Thrombin-induced chemotaxis and aggregation of neutrophils. Journal of cellular physiology.

[40] Stankova J, Rola-Pleszczynski M, D’Orleans-Juste P (1995). Endothelin 1 and thrombin synergistically stimulate IL-6 mRNA expression and protein production in human umbilical vein endothelial cells. Journal of cardiovascular pharmacology.

[41] Ueno A, Murakami K, Yamanouchi K, Watanabe M, Kondo T (1996). Thrombin stimulates production of interleukin-8 in human umbilical vein endothelial cells. Immunology.

[42] Demidova-Rice TN, Wolf L, Deckenback J, Hamblin MR, Herman IM (2012). Human platelet-rich plasma- and extracellular matrix-derived peptides promote impaired cutaneous wound healing *in vivo*. PloS one.

[43] Sheets AR, Massey CJ, Cronk SM, Iafrati MD, Herman IM (2016). Matrix- and plasma-derived peptides promote tissue-specific injury responses and wound healing in diabetic swine. Journal of translational medicine.

[44] Stiernberg J (2000). Acceleration of full-thickness wound healing in normal rats by the synthetic thrombin peptide, TP508. Wound repair and regeneration: official publication of the Wound Healing Society [and] the European Tissue Repair Society.

[45] Morihara K, Tsuzuki H, Oka T, Inoue H, Ebata M (1965). Pseudomonas Aeruginosa Elastase. Isolation, Crystallization, and Preliminary Characterization. The Journal of biological chemistry.

[46] Schmidtchen A (2000). Degradation of antiproteinases, complement and fibronectin in chronic leg ulcers. Acta dermato-venereologica.

[47] Vizcaino JA (2014). ProteomeXchange provides globally coordinated proteomics data submission and dissemination. Nature biotechnology.

[48] Vizcaino, J. A. *et al*. update of the PRIDE database and its related tools. *Nucleic acids research* **44**, 11033, 10.1093/nar/gkw880 (2016). 10.1093/nar/gkw880 PMC5159556 27683222

