# Supplementary material for: Proteolytic signatures define unique thrombin-derived peptides present in human wound fluid in vivo
Source: Sci Rep. 2017 Oct 13;7:13136. doi: 10.1038/s41598-017-13197-3 (PMC5640616; doi:10.1038/s41598-017-13197-3)
Supplement: Supplementary file 1 — Supplementary Information [file 41598_2017_13197_MOESM1_ESM.pdf]

SUPPLEMENTARY INFORMATION

PROTEOLYTIC SIGNATURES DEFINE UNIQUE THROMBIN-DERIVED PEPTIDES PRESENT IN HUMAN WOUND FLUID *IN VIVO*

Rathi Saravanan, Sunil S Adav, Yeu Khai Choong, Mariena J.A. van der Plas, Jitka Petrlova, Sven Kjellström, Siu Kwan Sze, Artur Schmidtchen

Figure S1

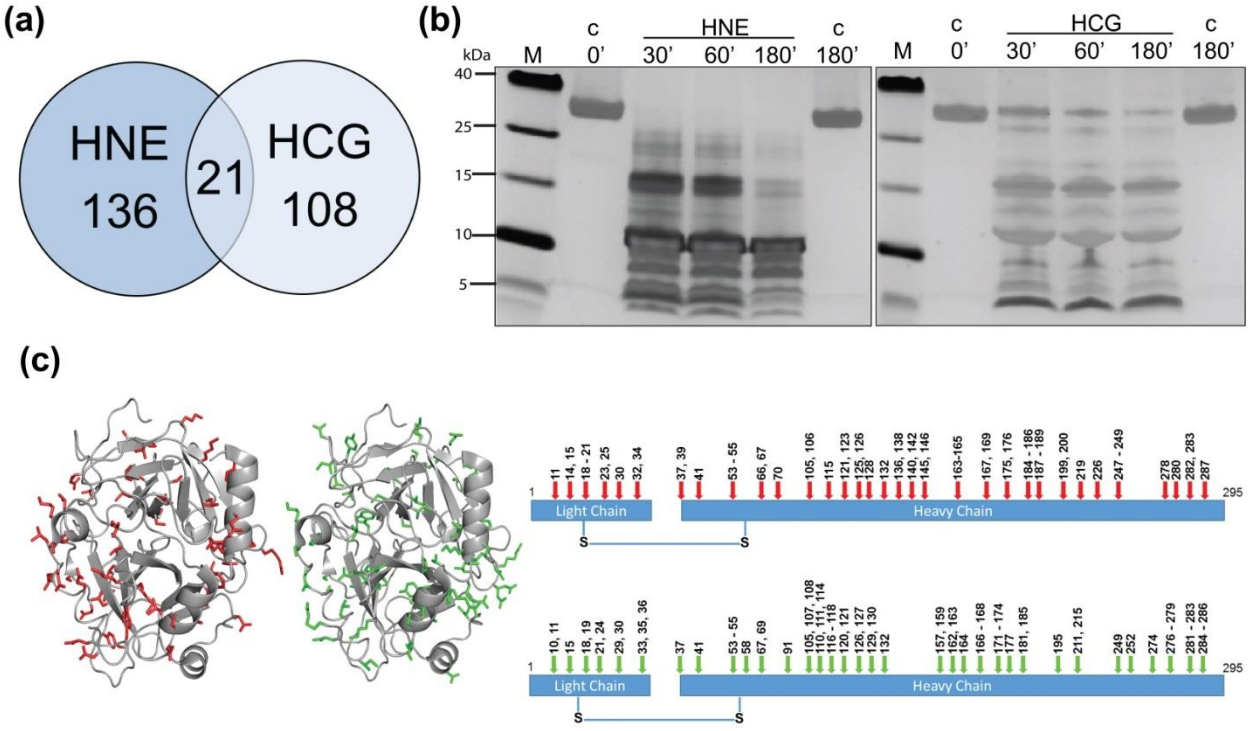

Figure S1: Proteolytic digestion of thrombin by human neutrophil elastase (HNE) and cathepsin G (HCG). (**a**) A Venn-diagram representing the complete degradome of thrombin digested with HNE and HCG. 21 fragments were found common to both human proteases studied. (**b**) Silver stain image of thrombin incubated with human proteases shows formation of distinct peptide digestion patterns. (**c**) 3D model and 2D representation of thrombin showing LC-MS/MS identified cleavage points for HNE (red) and HCG (green).

Figure S2

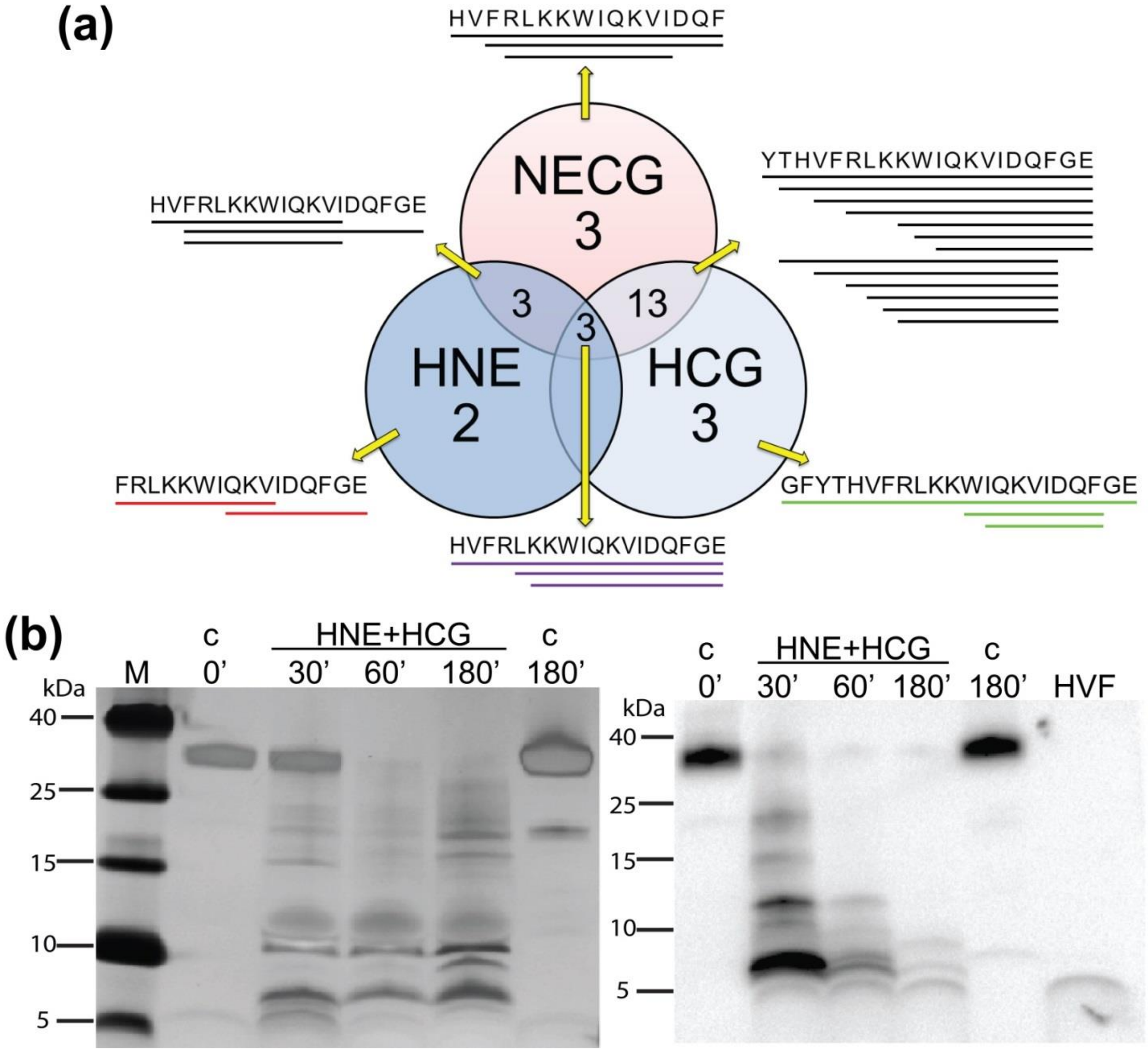

Figure S2: Combined proteolytic effects of HNE and HCG on thrombin. (**a**) Comparison of the digestion pattern of TCPs generated by HNE (red) and HCG (green) individually or in combination of HNE and HCG (black). Peptides identified common in all three *in vitro* digests is shown in purple (**b**) Silver stain (left) and western blot images (right) of thrombin incubated simultaneously with HNE and HCG at the indicated time points

Figure S3

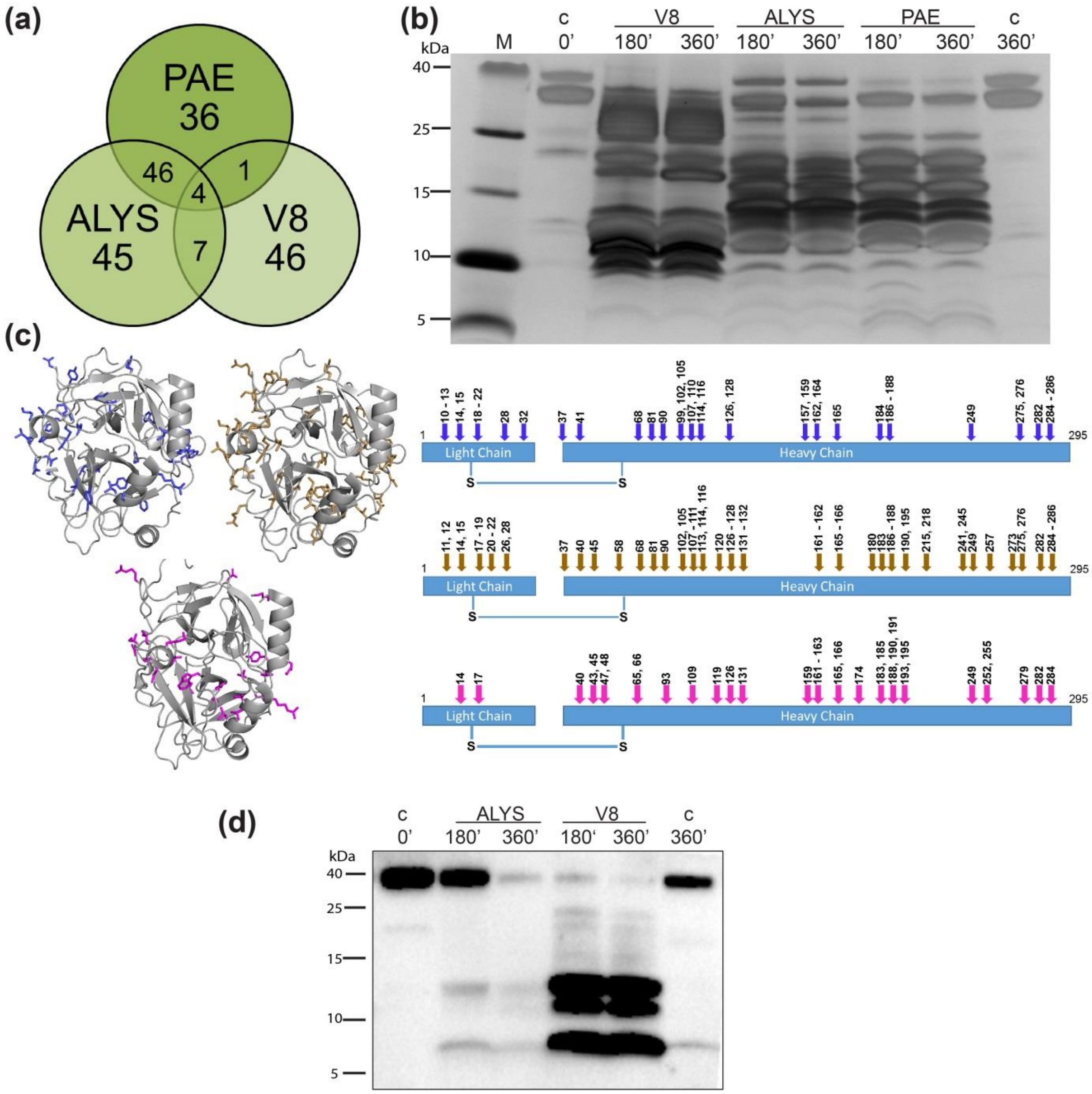

Figure S3. Proteolytic digestion of thrombin by bacterial proteases. (**a**) A Venn-diagram representing the complete degradome of thrombin with PAE, ALYS, and V8. The high number of common sequences between PAE and ALYS highlights the similarities in bacterial M4 peptidase group. (**b**) Silver stain image shows thrombin digestion patterns by PAE, ALYS and V8 at the indicated time points. (**c**) 3D model and 2D representation of thrombin showing LC-MS/MS identified cleavage points for PAE (blue), ALYS (brown) and V8 (magenta). **(d)** Western blot analysis of the thrombin digestion patterns by V8 and ALYS at different exposure times. TCPs generated by ALYS were unambiguously evident at higher exposure time of 20 secs.

Figure S4

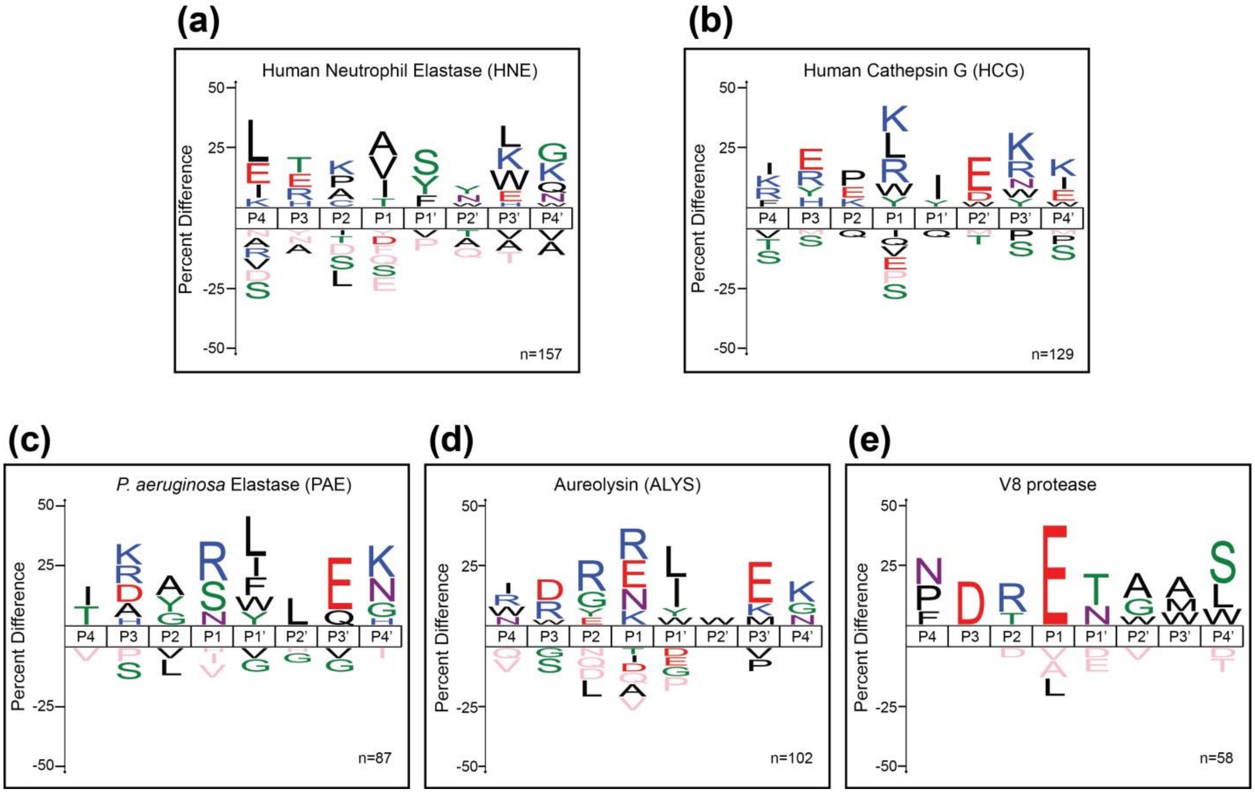

Figure S4. Substrate specificity profiling of the different human and bacterial proteases studied. IceLogos representing the P4 to P4' sites for (a) neutrophil elastase (b) cathepsinG (c) *Pseudomonas aeruginosa* elastase (d) Staphylococcus aureus aureolysin and (e) V8 proteases. Amino acids that are most frequently observed (above axis) and least frequently observed (below axis) are indicated. The color coding was set as default and the number of cleavage sites used to generate each IceLogo is provided at the bottom right-hand corner.

Table S1

Table S1: Sequences of total thrombin peptides generated common by human neutrophil elastase and cathepsin G

Sequences	Enzyme(s)		Mass (Da)		Cleaving position
	HNE	HCG	Theoretical	Experimental	
**HVFRLKKWIQKVIDQFGE**	+	+	2270.269	2270.332	T/H
**KKWIQKVIDQFGE**	+	+	1617.888	1617.967	L/K
**LKKWIQKVIDQFGE**	+	+	1730.972	1731.117	R/L
EDKTERELLESYIDGR	+	+	1951.949	1952.186	L/E
FEKKSLEDKTERELL	+	+	1863.994	1864.055	L/F
FEKKSLEDKTERELLE	+	+	1993.037	1993.262	L/F
FEKKSLEDKTERELLESY	+	+	2243.132	2243.194	L/F
FEKKSLEDKTERELLESYIDG	+	+	2528.265	2528.628	L/F
SLEDKTERELLESYIDGR	+	+	2152.065	2152.188	K/S
SLISDRWVL	+	+	1087.603	1087.603	A/S
YIHPRYNWRENLDRDIALMKLKKPV	+	+	3167.718	3167.871	I/Y
YNWRENLDRDIAL	+	+	1676.827	1676.839	R/Y
YNWRENLDRDIALM	+	+	1807.868	1807.950	R/Y
FEKKSLEDKTERELLESYIDGR	+	+	2684.366	2684.528	L/F
IVEGSDAEIGMSPWQVM	+	+	1879.833	1879.875	R/I
TANVGKGQPSVLQVV	+	+	1495.836	1495.904	W/T
YNWRENLDRDIA	+	+	1563.743	1563.754	R/Y
FEKKSLEDKTEREL	+	+	1750.910	1750.946	L/F
FRKSPQELL	+	+	1116.629	1116.629	L/F
LFRKSPQELL	+	+	1229.713	1229.730	M/L
YNWRENLDRD	+	+	1379.622	1379.673	R/Y

*Sequences highlighted in bold are thrombin C-terminal peptides (TCPs)

Table S2

Table S2: Sequences of total thrombin peptides identified common to the bacterial proteases studied.

Sequences	Enzyme(s)			Mass (Da)		Cleaving position
	PAE	ALYS	V8	Theoretical	Experimental	
**FYTHVFRLKKWIQKVIDQFGE**	+	+		2681.448	2681.599	G/F
**IQKVIDQFGE**	+	+		1175.619	1175.648	W/I
**WIQKVIDQFGE**	+	+		1361.698	1361.697	K/W
**YTHVFRLKKWIQKVIDQFGE**	+	+		2534.380	2534.638	F/Y
AASLLQAGYKGRVTGWGN	+	+		1847.964	1847.966	T/A
ANVGKGQPSVLQVVNLP	+	+		1718.968	1719.114	T/A
DKTERELLESYIDGR	+	+		1822.906	1823.261	S/L
EDKTERELLESYIDGR	+	+		1951.949	1952.186	L/E
FEKKSLEDKTERELLESYIDGR	+	+		2684.366	2684.528	L/F
FTENDLLVR	+	+		1105.577	1105.669	N/F
FTENDLLVRI	+	+		1218.661	1218.691	N/F
HSRTRYERNIEKISMLEKIYIHPRYN	+	+		3345.752	3345.978	K/H
ISMLEKIYIHPRYN	+	+		1775.939	1776.059	K/I
IVEGSDAEIGMSPWQ	+	+		1617.735	1617.732	R/I
IVEGSDAEIGMSPWQVM	+	+		1879.833	1879.875	R/I
IVEGSDAEIGMSPWQVMLFR	+	+		2264.097	2264.239	R/I
IVEGSDAEIGMSPWQVMLFRK	+	+		2392.192	2392.525	R/I
IVEGSDAEIGMSPWQVMLFRKSPQE	+	+		2833.378	2833.737	R/I
IVEGSDAEIGMSPWQVMLFRKSPQEL	+	+		2946.462	2946.454	R/I
KSLEDKTERELLESYIDGR	+	+		2280.160	2280.230	K/K
LEDKTERELLESYIDGR	+	+		2065.033	2065.166	S/L
LFEKKSLEDKTERELLES	+	+		2193.153	2193.278	P/L
LISDRWVLT	+	+		1101.618	1101.618	S/L
LLESYIDGR	+	+		1064.550	1064.582	E/L
LLQAGYKGRVTGWG	+	+		1504.815	1504.899	S/L
LLQAGYKGRVTGWGN	+	+		1618.858	1618.947	S/L
LLQAGYKGRVTGWGNLKET	+	+		2090.127	2090.246	S/L
LLYPPWDKNFTENDLLVR	+	+		2233.142	2233.443	C/L
LRPLFEKKSLEDKTERELLESYIDGR	+	+		3163.688	3164.103	G/L
MLEKIYIHPRYNWRENLDRDIA	+	+		2844.449	2844.448	S/M
NIEKISMLEKIYIHPR	+	+		1983.098	1983.199	R/N
NIEKISMLEKIYIHPRYN	+	+		2260.204	2260.304	R/N
NVGKGQPSVLQVVNLP	+	+		1647.931	1648.028	A/N
RPLFEKKSLEDKTERELLESYIDGR	+	+		3050.604	3051.183	L/R
SLEDKTERELLESYIDGR	+	+		2152.065	2152.188	K/S

Table S2 (continued)

Sequences	Enzyme(s)			Mass (Da)		Cleaving position
	PAE	ALYS	V8	Theoretical	Experimental	
TRYERNIEKISMLEKIYIHPR	+	+		2688.453	2688.819	R/T
TRYERNIEKISMLEKIYIHPRYN	+	+		2965.560	2965.681	R/T
VGKGQPSVLQVVNLP	+	+		1533.888	1533.974	N/V
WRENLDRDIAL	+	+		1399.721	1399.797	N/W
WRENLDRDIALMK	+	+		1658.856	1658.871	N/W
WRENLDRDIALMKLKKP	+	+		2125.183	2125.322	N/W
WRENLDRDIALMKLKKPVA	+	+		2295.289	2295.440	N/W
YERNIEKISMLEKIYIHPR	+	+		2431.305	2431.443	R/Y
YERNIEKISMLEKIYIHPRYN	+	+		2708.411	2708.594	R/Y
YNWRENLDRDIALMKLKKP	+	+		2402.289	2402.466	R/Y
YNWRENLDRDIALMKLKKPVA	+	+		2572.395	2572.509	R/Y
GSDAEIGMSPWQVMLFRKSPQE		+	+	2492.183	2492.729	E/G
IGMSPWQVMLFRKSPQE		+	+	2033.023	2033.163	E/I
KKSLEDKTERELLESYIDGR		+	+	2408.255	2408.330	E/K
LQAGYKGRVTGWGNLKE		+	+	1875.996	1876.428	L/L
TAASLLQAGYKGRVTGWGNLKE		+	+	2319.234	2319.327	E/T
TWTANVGKGQPSVLQ		+	+	1584.826	1585.120	E/T
YNWRENLDRDIALMK		+	+	1935.963	1935.961	R/Y
SPFNNRWYQ	+		+	1210.552	1210.551	K/S
**KWIQKVIDQFGE**	+	+	+	1489.793	1489.915	K/K
**LKKWIQKVIDQFGE**	+	+	+	1730.972	1731.117	R/L
LFEKKSLEDKTERELLESYIDGR	+	+	+	2797.450	2797.723	P/L
LLQAGYKGRVTGWGNLKE	+	+	+	1989.080	1989.431	S/L

*Sequences highlighted in bold are thrombin C-terminal peptides (TCPs)

Table S3

Table S3: Sequences of thrombin C-terminal peptides identified unique and common to acute wound fluid (AWF) and chronic wound fluid (CWF)

Sequences	Source(s)				Mass (Da)	
	HP	BP	AWF	CWF	Theoretical	Experimental
IQKVIDQFG			+		1046.565	1046.575
IDQFGE			+		707.302	707.313
VIDQFGE				+	806.370	806.379
KVIDQFGE			+	+	934.465	934.475
IQKVIDQF	+		+		989.544	989.554
FRLKKWIQKVIDQFGE	+			+	2034.131	2034.142
HVFRLKKWIQKVIDQFGE	+			+	2270.269	2270.267
FYTHVFRLKKWIQKVIDQFGE		+		+	2681.448	2681.446
KKWIQKVIDQFGE	+		+	+	1617.888	1617.892
QKVIDQFGE	+		+	+	1062.524	1062.531
LKKWIQKVIDQFGE	+	+	+	+	1730.972	1730.970
KWIQKVIDQFGE	+	+	+	+	1489.793	1489.793
WIQKVIDQFGE	+	+	+	+	1361.698	1361.698
IQKVIDQFGE	+	+	+	+	1175.619	1175.618

Table S4

Table S4: Peptide sequences identified in the study containing fragments of previously reported bio-active thrombin peptides

Sequences	Enzyme (s)					Mass (Da)	
	HNE	HCG	PAE	ALYS	V8	Theoretical	Experimental
FNPRTFGSGEADCGLRPLFEKKSLEDKTER**ELLESYIDGR**			+			4659.303	4659.615
GLRPLFEKKSLEDKTER**ELLESYIDGR**			+			3220.709	3220.898
GLRPLFEKKSLEDKTER**ELLESY**		+				2779.476	2780.182
LRPLFEKKSLEDKTER**ELLESYIDGR**			+	+		3163.688	3164.103
LRPLFEKKSLEDKTER**ELLESYI**	+					2835.538	2836.125
LRPLFEKKSLEDKTER**ELLESY**		+				2722.454	2722.676
LRPLFEKKSLEDKTER**ELLES**	+					2559.391	2559.809
RPLFEKKSLEDKTER**ELLESYIDGR**			+	+		3050.604	3051.183
PLFEKKSLEDKTER**ELLESYIDGR**			+			2894.503	2894.998
LFEKKSLEDKTER**ELLESYIDGR**IVEG			+			3195.666	3196.674
LFEKKSLEDKTER**ELLESYIDGR**	+		+	+	+	2797.450	2797.723
LFEKKSLEDKTER**ELLESYIDG**			+			2641.349	2641.585
LFEKKSLEDKTER**ELLESY**			+			2356.216	2356.513
LFEKKSLEDKTER**ELLES**	+		+	+		2193.153	2193.278
LFEKKSLEDKTER**ELLE**			+			2106.121	2106.141
FEKKSLEDKTER**ELLESYIDGR**IVEG		+				3082.582	3082.578
FEKKSLEDKTER**ELLESYIDGR**IV	+					2896.518	2896.616
FEKKSLEDKTER**ELLESYIDGR**I	+					2797.450	2797.953
FEKKSLEDKTER**ELLESYIDGR**	+	+	+	+		2684.366	2684.528
FEKKSLEDKTER**ELLESYIDG**	+	+				2528.265	2528.628
FEKKSLEDKTER**ELLESYID**		+				2471.243	2471.920
FEKKSLEDKTER**ELLESYI**	+					2356.216	2356.274
FEKKSLEDKTER**ELLESY**	+	+	+			2243.132	2243.194
FEKKSLEDKTER**ELLES**	+					2080.069	2080.106
FEKKSLEDKTER**ELLE**	+	+				1993.037	1993.262
FEKKSLEDKTER**ELL**	+	+				1863.994	1864.055
FEKKSLEDKTER**EL**	+	+				1750.910	1750.946
KKSLEDKTER**ELLESYIDGR**				+	+	2408.255	2408.330
KKSLEDKTER**ELLESYIDG**				+		2252.154	2252.583
KKSLEDKTER**ELLESY**				+		1967.021	1967.116
KSLEDKTER**ELLESYIDGR**		+	+	+		2280.160	2280.230
KSLEDKTER**ELLESYI**	+					1952.010	1952.008
KSLEDKTER**ELLESY**		+				1838.926	1839.068
SLEDKTER**ELLESYIDGR**	+	+	+	+		2152.065	2152.188

Table S4 (continued)

Sequences	Enzyme (s)					Mass (Da)	
	HNE	HCG	PAE	ALYS	V8	Theoretical	Experimental
SLEDKTER**ELLESYI**	+					1823.915	1823.913
SLEDKTER**ELLESY**		+				1710.831	1710.844
SLEDKTER**ELLES**	+					1547.768	1547.767
SLEDKTER**ELLE**		+				1460.736	1460.782
LEDKTER**ELLESYIDGR**			+	+		2065.033	2065.166
LEDKTER**ELLESYID**G			+			1908.932	1909.577
LEDKTER**ELLESYID**			+			1851.910	1852.110
LEDKTER**ELLESYI**	+					1736.883	1736.884
LEDKTER**ELLESY**				+		1623.799	1623.797
LEDKTER**ELLES**			+			1460.736	1460.736
EDKTER**ELLESYIDGR**	+	+	+	+		1951.949	1952.186
EDKTER**ELLESYI**	+					1623.799	1623.797
EDKTER**ELLESY**		+				1510.715	1510.784
DKTER**ELLESYIDGR**			+	+		1822.906	1823.261
KTER**ELLESYIDGR**	+					1707.879	1708.008
TER**ELLESYIDGR**		+				1579.784	1579.784
TER**ELLESY**		+				1138.551	1138.836
ER**ELLESYIDGR**	+					1478.737	1478.737
ER**ELLESYI**	+					1150.587	1150.587
R**ELLESYIDGR**				+		1349.694	1349.755
**LLESYIDGR**			+	+		1064.550	1064.582
TDNMFC**AGYKPDEGKRGDACEGDSGGPFV**MKSPFNNRWYQ				+		4588.957	4588.789
DNMFC**AGYKPDEGKRGDACEGDSGGPFV**MK	+					3294.384	3296.404
**YKPDEGKRGDACEGDSGGPFV**MK	+					2499.116	2499.111
